# Supplementary material for: Empirical evidence on factors influencing farmers’ administrative burden: A structural equation modeling approach
Source: PLoS One. 2020 Oct 30;15(10):e0241075. doi: 10.1371/journal.pone.0241075 (PMC7598450; doi:10.1371/journal.pone.0241075)
Supplement: S1 Table — (DOCX) [file pone.0241075.s003.docx]

**S1 Table. Direct effects of the (causal) structural model (unstandardized coefficients).**

| **Path** | **Model 1**  **SEM** | **Model 2**  **SEM** | **Model 3**  **SEM** |
| --- | --- | --- | --- |
| Knowledge level 🡪 administrative burden (H1) | -0.148  (0.357) | -0.586  (0.368) | -0.586  (0.368) |
| Compliance costs 🡪 administrative burden (H2) | 1.539***  (0.251) | 1.695***  (0.242) | 1.695***  (0.242) |
| Psychological costs 🡪 administrative burden (H3) | 0.318**  (0.142) |  |  |
| Administrative burden 🡪 psychological costs (H3a) |  | 0.147**  (0.062) |  |
| Administrative burden ↔ psychological costs (H3b) |  |  | 0.096**  (0.046) |
| Knowledge level 🡪 compliance costs (H4) | -0.057  (0.155) | -0.057  (0.155) | -0.057  (0.155) |
| Knowledge level 🡪 psychological costs (H5) | -1.378***  (0.412) | -1.291***  (0.382) | -1.378***  (0.412) |
| Compliance costs 🡪 psychological costs (H6) | 0.490***  (0.103) | 0.241  (0.120) | 0.490***  (0.103) |
| **Comparative model fit criteria** |  |  |  |
| AIC | 24,933 | 24,933 | 24,933 |
| BIC | 25,129 | 25,129 | 25,129 |
| Likelihood-ratio test | -12,421 | -12,421 | -12,421 |
| **Overall model fit criteria** |  |  |  |
| χ^2^ p-value | 0.000 | 0.000 | 0.000 |
| RMSEA | 0.069 | 0.069 | 0.069 |
| CFI | 0.908 | 0.908 | 0.908 |
| SRMR | 0.066 | 0.066 | 0.066 |

* p ≤ 0.1; ** p ≤ 0.05; *** p ≤ 0.01.

Standard errors based on Observed Information Matrix (OIM) in parentheses.
