# Supplementary material for: Empirical evidence on factors influencing farmers’ administrative burden: A structural equation modeling approach
Source: PLoS One. 2020 Oct 30;15(10):e0241075. doi: 10.1371/journal.pone.0241075 (PMC7598450; doi:10.1371/journal.pone.0241075)
Supplement: S2 Table — (DOCX) [file pone.0241075.s004.docx]

**S2 Table. Indirect and total effects of the (causal) structural model**

**(unstandardized coefficients).**

| **Indirect effects** | **Model 1**  **SEM** | **Model 2**  **SEM** | **Model 3**  **SEM** |
| --- | --- | --- | --- |
| Knowledge level 🡪 administrative burden  Mediator variable: compliance costs | -0.088  (0.240) | -0.097  (0.264) | -0.097  (0.264) |
| Knowledge level 🡪 administrative burden  Mediator variable: psychological costs | -0.438*  (0.355) |  |  |
| Compliance costs 🡪 administrative burden  Mediator variable: psychological costs | 0.156**  (0.062) |  |  |
| Compliance costs 🡪 psychological costs  Mediator variable: administrative burden |  | 0.249**  (0.107) |  |
| **Total effects** | **Model 1**  **SEM** | **Model 2**  **SEM** | **Model 3**  **SEM** |
| Knowledge level 🡪 administrative burden  Mediator variable: compliance costs | -0.237  (0.366) | -0.684*  (0.373) | -0.684*  (0.373) |
| Knowledge level 🡪 administrative burden  Mediator variable: psychological costs | -0.586  (0.368) |  |  |
| Compliance costs 🡪 administrative burden  Mediator variable: psychological costs | 1.695***  (0.242) |  |  |
| Compliance costs 🡪 psychological costs  Mediator variable: administrative burden |  | 0.490***  (0.103) |  |

* p ≤ 0.1; ** p ≤ 0.05; *** p ≤ 0.01.

Standard errors based on delta method in parentheses.
