# Supplementary material for: Empirical evidence on factors influencing farmers’ administrative burden: A structural equation modeling approach
Source: PLoS One. 2020 Oct 30;15(10):e0241075. doi: 10.1371/journal.pone.0241075 (PMC7598450; doi:10.1371/journal.pone.0241075)
Supplement: S3 Table — (DOCX) [file pone.0241075.s005.docx]

**S3 Table: The measurement models (unstandardized coefficients).**

| **Latent variable** | **Observed variable** | **Model 1**  **SEM** | | **Model 2**  **SEM** | | **Model 3**  **SEM** | |
| --- | --- | --- | --- | --- | --- | --- | --- |
|  |  | **Coeff.** | **Std.Err.** | **Coeff.** | **Std.Err.** | **Coeff.** | **Std.Err.** |
| Administrative burden | $y_{1}$ | 1.000 |  | 1.000 |  | 1.000 |  |
|  | $y_{2}$ | 0.752 | 0.066 | 0.752 | 0.066 | 0.752 | 0.066 |
| Compliance costs | $y_{3}$ | 1.375 | 0.187 | 1.375 | 0.187 | 1.375 | 0.187 |
|  | $y_{4}$ | 1.000 |  | 1.000 |  | 1.000 |  |
|  | $y_{5}$ | 1.237 | 0.177 | 1.237 | 0.177 | 1.237 | 0.177 |
| Psychological costs | $y_{6}$ | 1.000 |  | 1.000 |  | 1.000 |  |
|  | $y_{7}$ | 1.688 | 0.211 | 1.688 | 0.211 | 1.688 | 0.211 |
|  | $y_{8}$ | 2.001 | 0.248 | 2.001 | 0.248 | 2.001 | 0.248 |
|  | $y_{9}$ | 1.081 | 0.186 | 1.081 | 0.186 | 1.081 | 0.186 |
| Knowledge level | $x_{1}$ | 1.000 |  | 1.000 |  | 1.000 |  |
|  | $x_{2}$ | 3.498 | 1.001 | 3.498 | 1.001 | 3.498 | 1.001 |
|  | $x_{3}$ | 5.576 | 1.547 | 5.576 | 1.547 | 5.576 | 1.547 |
|  | $x_{4}$ | 4.528 | 1.218 | 4.528 | 1.218 | 4.528 | 1.218 |

Note: All coefficients are significantly different from zero on the 99 percent confidence interval
