# Supplementary material for: Empirical evidence on factors influencing farmers’ administrative burden: A structural equation modeling approach
Source: PLoS One. 2020 Oct 30;15(10):e0241075. doi: 10.1371/journal.pone.0241075 (PMC7598450; doi:10.1371/journal.pone.0241075)
Supplement: S4 Table — (DOCX) [file pone.0241075.s006.docx]

**S4 Table: The measurement models (unstandardized coefficients).**

| **Latent variable** | **Observed variable** | **Model 1**  **GSEM** | | **Model 2**  **GSEM** | | **Model 3**  **GSEM** | |
| --- | --- | --- | --- | --- | --- | --- | --- |
|  |  | **Coeff.** | **Std.Err.** | **Coeff.** | **Std.Err.** | **Coeff.** | **Std.Err.** |
| Administrative burden | $y_{1}$ | 1.000 |  | 1.000 |  | 1.000 |  |
|  | $y_{2}$ | 0.779 | 0.139 | 0.779 | 0.139 | 0.779 | 0.139 |
| Compliance costs | $y_{3}$ | 0.721 | 0.118 | 0.718 | 0.118 | 0.720 | 0.118 |
|  | $y_{4}$ | 1.000 |  | 1.000 |  | 1.000 |  |
|  | $y_{5}$ | 0.825 | 0.133 | 0.824 | 0.133 | 0.825 | 0.133 |
| Psychological costs | $y_{6}$ | 0.450 | 0.055 | 0.450 | 0.055 | 0.450 | 0.055 |
|  | $y_{7}$ | 1.000 |  | 1.000 |  | 1.000 |  |
|  | $y_{8}$ | 1.282 | 0.177 | 1.280 | 0.177 | 1.249 | 0.168 |
|  | $y_{9}$ | 0.446 | 0.053 | 0.446 | 0.053 | 0.446 | 0.053 |
| Knowledge level | $x_{1}$ | 1.000 |  | 1.000 |  | 1.000 |  |
|  | $x_{2}$ | 11.986 | 3.224 | 11.987 | 3.226 | 11.999 | 3.223 |
|  | $x_{3}$ | 7.858 | 1.919 | 7.870 | 1.924 | 7.858 | 1.919 |
|  | $x_{4}$ | 4.909 | 1.245 | 4.916 | 1.245 | 4.909 | 1.245 |

Note: All coefficients (loadings) are significantly different from zero on the 99 percent confidence interval.
